# Inter-Segmental Coordination Pattern in Patients with Anterior Cruciate Ligament Deficiency during a Single-Step Descent

**DOI:** 10.1371/journal.pone.0149837

**Published:** 2016-02-22

**Authors:** Mohammadreza Nematollahi, Mohsen Razeghi, Sina Mehdizadeh, Hamidreza Tabatabaee, Soraya Piroozi, Zahra Rojhani Shirazi, Ali Rafiee

**Affiliations:** 1 Department of Physiotherapy, School of Rehabilitation Sciences, Shiraz University of Medical Sciences, Shiraz, Iran; 2 Biomechanics and Sports Engineering Group, Faculty of Biomedical Engineering, Amirkabir University of Technology, Tehran, Iran; 3 Epidemiology Department, School of Health, Shiraz University of Medical Sciences, Shiraz, Iran; 4 Electrical Department, Kazeroon Branch, Azad University, Kazeroon, Iran; West Virginia University, UNITED STATES

## Abstract

Anterior cruciate ligament injury is a debilitating pathology which may alter lower limb coordination pattern in both intact and affected lower extremities during activities of daily living. Emerging evidence supports the notion that kinematic variables may not be a good indicator to differentiate patients with anterior cruciate ligament deficiency during step descent task. The aim of the present study was to examine alterations in kinematics as well as coordination patterns and coordination variability of both limbs of these patients during a single step descent task. Continuous relative phase technique was used to measure coordination pattern and coordination variability between a group of anterior cruciate ligament deficient (n = 23) and a healthy control group (n = 23). A third order polynomial Curve fitting was utilized to provide a curve that best fitted to the data points of coordination pattern and coordination variability of the healthy control group. This was considered as a reference to compare to that of patient group using nonlinear regression analysis. The results of the present study demonstrated an altered coordination pattern of the supporting shank-thigh and the stepping foot-shank couplings in anterior cruciate ligament deficient subjects. It was further noticed that there was an increased coordination variability in foot-shank and shank-thigh couplings of both supporting and stepping legs. There was no significant difference in the hip, knee and ankle joints kinematics in either side of these patients. Anterior cruciate ligament deficient individuals showed altered strategies in both intact and affected legs, with increased coordination variability. Kinematic data did not indicate any significant difference between the two groups. It could be concluded that more sophisticated dynamic approach such as continuous relative phase would uncover discrepancies between the healthy and anterior cruciate ligament deficient individuals.

## Introduction

Deficiency of anterior cruciate ligament (ACL) not only reduces the stability of the knee joint but also compromises lower limb coordination during functional tasks [1,2]. Previous studies demonstrated alterations in the movement characteristics of both the affected and unaffected sides in these patients [3,4]. For example, it is reported that the lower limb flexion/extension moment of ACL deficient (ACLD) subjects was substantially different from those of normal population. This was the case for both lower limbs and was spared from the kinematics of movement [5]. Therefore, such a deficiency has tremendous effects not only on the ipsilateral side but also on other joints or segments in the countralateral side [3,5–7]. As such, enough evidence exists to show that this local injury could modify strategies in a more global fashion. Therefore, in the case of unilateral ACL injuries, it is quite possible to observe altered coordination patterns in both limbs with or without changes in the kinematics of movement.

Considering that the interlimb or intralimb coordination entails the interaction of many segments and muscles, examining the kinematics/kinetics of single joints may not be a good representation of the coordination among different joints [8–10]. Sophisticated dynamical system approaches have been developed to quantitatively measure coordination and provide higher dimensional information. One such method is the Continuous Relative Phase (CRP) which incorporates the velocity and position of two segments of interest [9,11]. It is suggested that this approach fully explains the dynamics of the system at the segmental level [8].

Studies on single joints’ kinematics and kinetics have frequently appeared in the literature [12,13]. However, such an approach provides lower dimensional information and cannot be a good representation of segmental coordination and therefore, cannot provide a full description of the pathology [11]. Here we used a dynamical system approach to investigate the coordination pattern of ACLD subjects in a functional task: stepping down. Since this movement entails intra-limb coordination, this provided us with a good opportunity to examine how the deficiency in a supportive structure affects the whole patterns of coordination. It is suggested that in activities such as stepping down the kinematics are very likely not to be affected [5]. Therefore, verifying step descent task would help us to examine coordination even if the kinematics is unaltered. On the other hand, it has been repeatedly shown that variability substantially increases in pathologic conditions and is an indicator of adaptation of motor control system dealing with challenging situations [14,15]. Therefore, examining variability in this functional task could provide valuable insight on the pathophysiology of the ACL deficiency. Here again, instead of evaluating the variability of single segments, coordination variability has been examined.

We investigated the biomechanics of a simple, yet challenging functional task of stepping down to verify possible changes in the kinematics and coordination of lower limbs with the assumption that these two entities might not be affected in the same manner. The results demonstrated that kinematic data do not show any significant difference between the two groups. However, using continuous relative phase, we are able to distinguish patients with ACLD knee from healthy control group.

## Materials and Methods

### Participants

From 47 potentially-eligible patients, 23 volunteered to participate in the present study. They were recruited from the surgery waiting list of the patients whose injury occurred 6 to 12 months earlier. Inclusion criteria were: (a) non-operated, chronic (more than 6 months after injury), right-sided, and complete ACL rupture with or without meniscal injury, diagnosed by MRI and clinical examination; (b) pain less than grade three according to visual analogue scale (0–10) at the time of examination; not to interfere in task performance, and (c) no history of any other musculoskeletal or neurological injury. Patients with ACLD knee differ functionally based on ability to cope with their injury. In this study, ACLD subjects were non-coper based on criteria established in the literature [16], i.e. patients were recruited if they had at least one of the following criteria:

Experienced two or more episodes of giving way since injury, global rating of function rated less than 60% of that of pre-injury level, and acquire less than 60% of that of the intact side in 6m timed hop test.

The control group consisted of 23 healthy male subjects. They were university students who volunteered to participate in the study. They were right-leg dominant as the patient group. The two groups were matched according to age, mass, height, and activity level (Table 1). In the present study, right and left legs of the healthy subjects were compared to right and left legs of the ACLD subjects, respectively. In order to minimize the effect of activity level on the study results, Tegner activity scale was utilized to compare the activity level of the two groups prior to ACL injury [17]. All participants signed an informed consent form before participating in the study. This study has been approved ethically by the Ethics Committee of Shiraz University of Medical Science (reference number: CT-91-6336).

**Table 1:** Descriptive characteristics of ACLD subjects and healthy control group.

Item	ACLD (n = 23)	Control (n = 23)
Age(y)	27.56±5.29	25.65±5.14
Height(cm)	176.17±4.72	177.52±6.43
Mass (Kg)	76.04±8.55	74.39±11.17
Activity level (0–10)	5.76±1.77	5.52±1.59
Time since injury (month)	8.02±2.28	-
Preferred speed (m/s)	0.38±0.04	0.37±0.03

ACLD = Anterior cruciate ligament deficient

### Procedure

**Fig 1:**
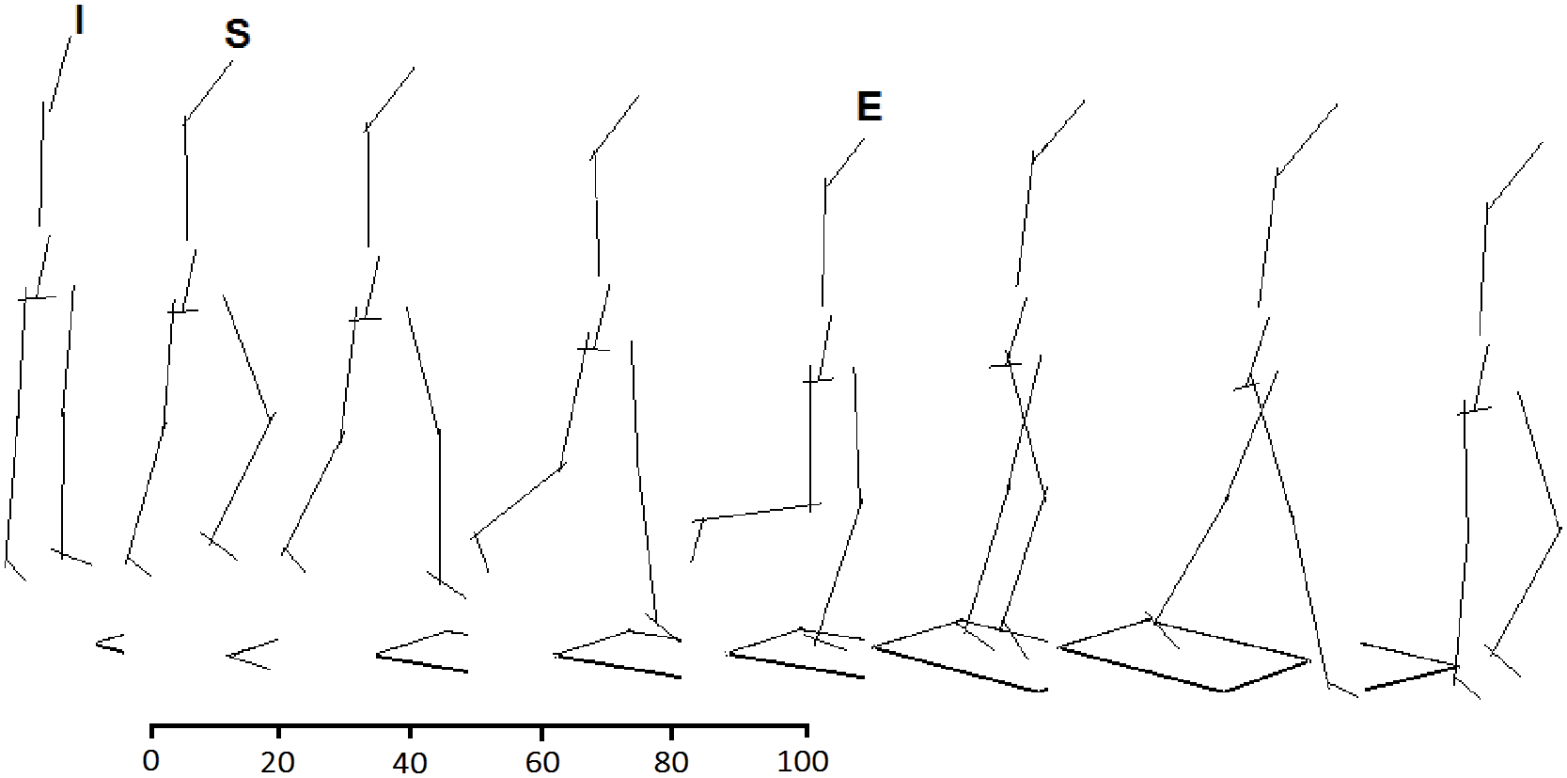
Step cycle. The supporting limb (right limb) is on the step and the stepping limb (left limb) touches the ground. I: initial position, S: start-point (maximum mediolateral position of the center of mass), E: end-point (minimum vertical position of the center of mass). The distance between the points S and E considered as task cycle and interpolated to 100 points.

Laboratory set-up “S1 Fig, Fig 1” consisted of a 21 cm height custom-made step, the highest entitled height of step proposed by International Code Council 2003 [18], in a 5 meter walkway. The laboratory space was surrounded by eight Qualysis motion analysis system cameras (Qualysis Track Manager System version 2.9, Gothenburg, Sweden) which were used to capture the coordinates of passive reflective markers at 60 Hz. The calibration markers were mounted to the specified landmarks based on Visual 3D guidelines as follows: anterolateral and posterolateral of the head, acromions, iliac crests, anterior and posterior iliac spines, right and left greater trochanters, medial and lateral epicondyles of femur, medial and lateral maleoli, both calcanei, heads of first and fifth metatarsals, and bases of the fifth metatarsals. Seven tracking markers were installed on the thorax to track this segment. Four clusters were used as tracking markers to track thighs and shanks. The head and trunk were built for accurate tracing of the center of mass.

Subjects placed their feet in the specified position on the step, with weight distributed evenly between the two feet. Initial position was monitored using a vertical bar with two horizontal pointers; one along maximum thoracic curvature and the other aligned with the point between the two posterior superior iliac spines to ensure consistent initial condition for all trials [19]. They were also asked to hold their head upright and look at a specified marker installed on the front wall a five-meter distance prior to starting descending step. In addition to dynamic trials, a standing static trial was recorded to establish neutral anatomical position.

Prior to each trial, subjects were asked to assume the same initial position. This position was monitored by two experimenters to ensure that initial position was reproducible across successive trials.

All participants were instructed to cross their arms against their chest in order to not utilize the upper body in balance compensations and also not to mask reflective markers while performing the task [20]. All subjects were instructed to land with their left (stepping) foot, while their right (supporting) leg was on the step to control lowering of the body mass. Kinematic data were recorded for seven seconds.

In this study, a single step which was employed previously was used to test our hypothesis [16,20,21]. All participants were requested to step down at their preferred speed and to continue to the end of the walkway “Fig 1”. Each subject completed a minimum of twenty trials of the step-descent task. Twenty trials with good marker visibility were selected for further analysis. Coordination pattern, coordination variability and ankle, knee and hip joints kinematics were considered as dependant variables. Likewise, group (ACLD and control) was considered as independent variable.

### Data analysis

After data collection, body was modeled with a nine-segment model in the Visual 3D software consisting of the following segments: foot, shank, and thigh of both the supporting and stepping legs, pelvis, trunk, and head-neck segments in the sagittal plane. Using Visual 3D software, marker’s coordinates were filtered with a fourth-order Butterworth filter with the cut-off frequency of 6 Hz. Previous research showed that averaging variability within a specific part of a task provides more sensitive detection of inter-subject disparities [22–24]. In the present study, frames associated with maximum mediolateral and minimum vertical positions of the center of mass were established using center of mass trajectory for start-point and end-point of the task, respectively. The angle data between these two points were then interpolated using a cubic spline technique [9,25,26]. The extracted period corresponds mainly to controlled lowering and stepping phases of the step-descent task [27].

To determine coordination patterns, Continuous Relative Phase method was implemented.

**Fig 2:**
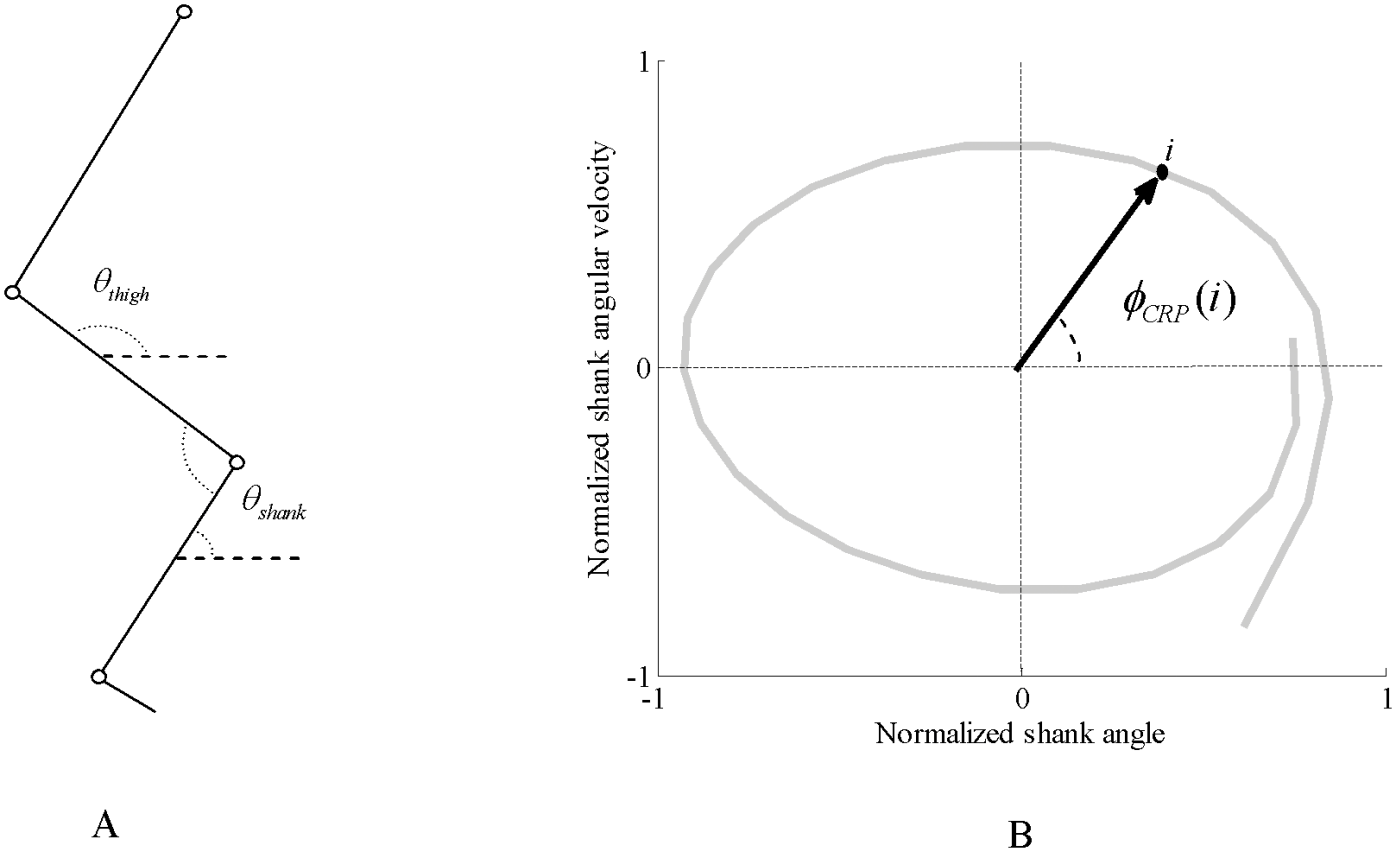
(A) Representation of segment and joint angles. θ_*thigh*_ and θ_*shank*_ are thigh and shank angles, respectively, (B) phase portrait of the shank to calculate shank phase angle. Such phase portrait was also constructed for thigh. Continuous relative phase was calculated by subtracting shank phase angle from thigh phase angle.

In this method, the relative phase is first calculated by plotting the position of one joint or segment with respect to angular velocity of that joint or segment in a phase plane [11]. Continuous relative phase was then calculated for the foot-shank and shank-thigh couplings in the sagittal plane by subtracting the relative phase of distal segment from proximal segment [10] “Fig 2”.

To calculate the CRP, phase angle (*∅*_*CRP*_) was first determined using Eq (1)

$$\phi_{CRP}\left(i\right)={\text{tan}}^{-1}\left(\omega_{norm}\left(i\right)/\theta_{norm}\left(i\right)\right);$$ (1)

Indeed, phase angle is the angle between a line connecting the origin to a data point, *i* and horizontal line in the phase plot of *θ*_*norm*_ and ω_*norm*_ [11].

Continuous relative phase was then calculated using Eq (2)

$$\Delta \phi_{CRP\;sh/th}\left(i\right)=abs\left(\phi_{CRP\;sh}\left(i\right)-\phi_{CRP\;th}\left(i\right)\right)$$ (2)

Where Δ*∅*_*CRP*,*sh/th*_(*i*) is the shank- thigh CRP at point *i*. *∅*_*CRP*,*sh*_ and *∅*_*CRP*,*th*_ are also shank and thigh phase angles, respectively, where *abs* represents absolute value. To eliminate discontinuities in continuous relative phase curves, values greater than 180 degrees were subtracted from 360, which resulted in continuous relative phases between 0 and 180 degrees [9]. The greater the continuous relative phase value, the more anti-phase segments and vice versa. Anti-phase relationship indicates that the two segments are moving in the opposite direction and in-phase relationship denotes the two segments are moving in the same direction. The reversals demonstrate changes in coordination dynamic [8].

The continuous relative phase variability was defined as the between-cycle standard deviation of continuous relative phase curve data points for each participant and was averaged over the cycle (over all data points *i*). Kinematics of the hip, knee and ankle of both the supporting and stepping legs during the early phase and at initial contact of the step-descent task was calculated to determine whether discrete kinematics demonstrate differences between the two groups.

A third-order polynomial curve fit with cubic model was utilized to construct a curve that best approximated our data points of the control group. Then, the coefficients were compared to that of patient group to verify the differences between the two groups. Cubic model was used as it was the model that competently fit to our data points. No additional processing was applied on our data points. Nonlinear regression analysis was used to compare coordination pattern and variability between the two groups. The descriptive characteristics (age, height, weight and activity level) between the two groups were compared using an independent *t-*test.

## Results

### Supporting leg

**Fig 3:**
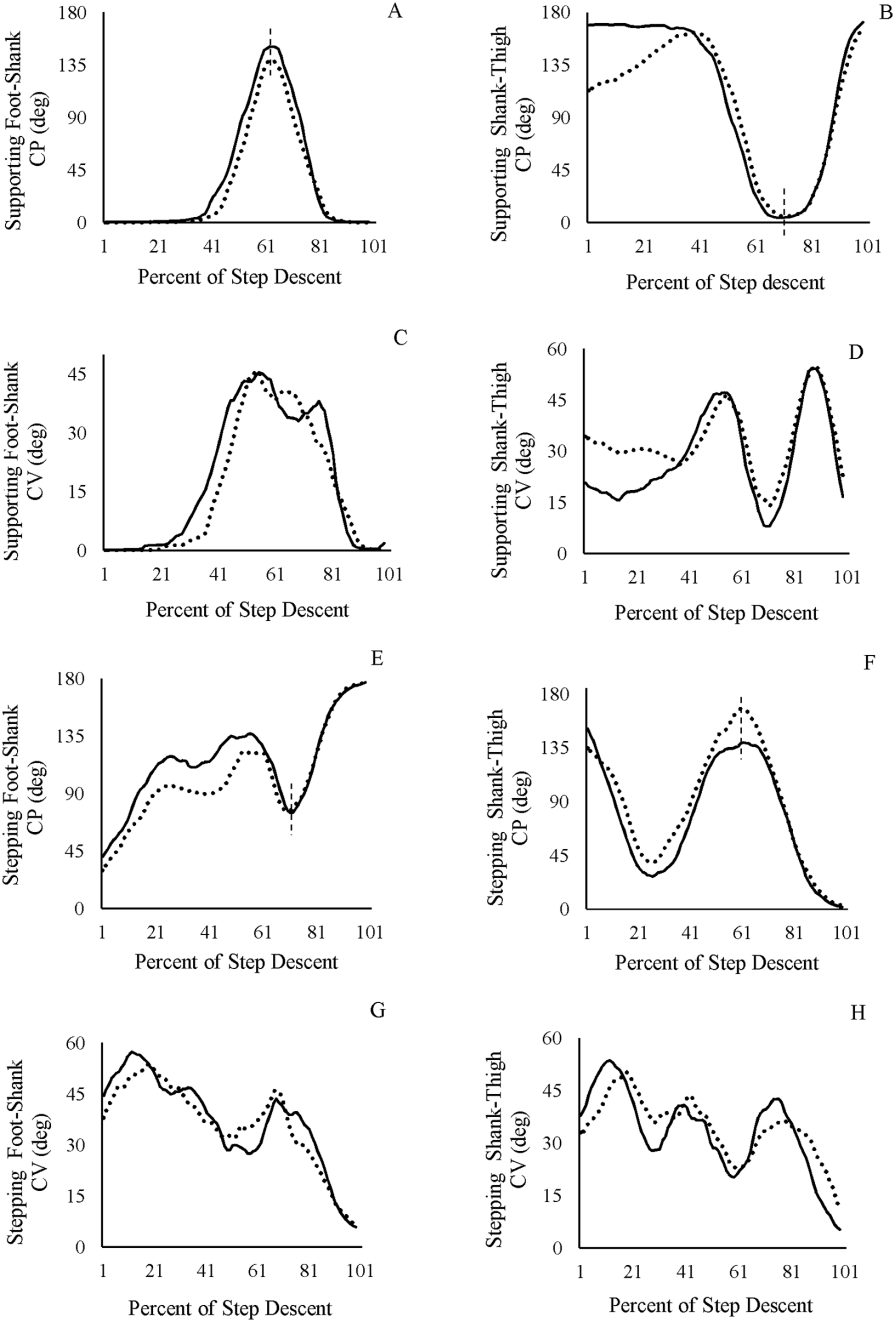
Mean ensemble coordination pattern and variability in foot-shank and shank-thigh couplings during a single-step descent. The bold line represents the healthy and the dotted line represents the ACLD subjects. CP: coordination pattern, CV: coordination variability. Values are in degree. Vertical dashed lines represent the location of reversals.

Based on the coefficients of the nonlinear regression analysis, no difference was seen in the coordination pattern of the supporting foot-shank coupling between the two groups. In the first 30 percent and last 20 percent of the task period, the values of foot-shank coupling are very close to zero in both groups, which suggests the two segments exhibit an in-phase relationship. With regard to minimums and maximums of the relative phase curve, the reversal was similar between the two groups “Fig 3A”.

There was a difference in the coordination pattern of the supporting shank-thigh between the healthy and ACLD subjects. Patients demonstrated more in-phase relationship in the early portion of the task. Nevertheless, the healthy control group had an anti-phase relationship. After 30 percent of the task period, the two groups demonstrated similar pattern with similar minimums and maximums “Fig 3B”.

There were also dissimilarities in the coordination variability of the supporting foot-shank and shank-thigh couplings. Coordination variability of the supporting foot-shank coupling was higher around the initial contact phase of the step-descent “Fig 3C”. For the shank-thigh coupling, coordination variability was higher in the first half of the task period. It also demonstrated higher coordination variability around initial contact “Fig 3D”.

### Stepping leg

For the stepping leg, there was a difference in the coordination pattern of the foot-shank couplings between the two groups. The ACLD subjects had a more in-phase relationship, specifically before initial contact. Moreover, while the reversals were similar, magnitudes of minimums and maximums differed. After initial contact, similar pattern is discernible between the two groups “Fig 3E”.

The stepping shank-thigh coordination pattern did not show difference between the two groups “Fig 3F”. This is justified based on the coefficients of the nonlinear regression analysis.

There were dissimilarities between the two groups in the coordination variability of the stepping foot-shank and shank-thigh couplings. Coordination variability of the foot-shank and shank-thigh couplings was higher around initial contact in the ACLD subjects. Generally, in both groups the amount of variability showed a declining trend as the swing-leg was entering the stance phase “Fig 3G and 3H”.

Kinematics of the hip, knee and ankle of both the supporting and stepping legs during the early phase and at initial contact of the step-descent task demonstrated no significant difference between the two groups. For example, the supporting knee flexion angle at early phase and stepping ankle plantar flexion angle at initial contact of the step-descent task were 26.78±6.84 and 28.05±6.68 in the healthy and 26.91±7.78 and 29.25±5.71 in the ACLD subjects respectively. (P value = 0.95 and 0.51, respectively) (Effect Size = 0.017 and 0.19 respectively). Likewise, the supporting knee flexion range of motion was 60.63±7.12 and 58.70±9.24 in healthy and ACLD subjects, (P value = 0.43) (Effect Size = 0.23). Furthermore, the average knee flexion angle of the supporting knee at initial contact was 49.70±9.39 and 47.02±7.47 in healthy and ACLD subjects respectively, (P value = 0.29) (Effect Size = 0.31).

## Discussion

The aim of the present study was to evaluate how the coordination patterns and kinematics of the ACLD subjects may be affected during a single-step descent. The results indicated altered relative phase dynamics in ACLD subjects. These findings confirm the results of previous investigations, which denoted strategy modification after ACL deficiency during different activities [1,5,6,16,28,29]. However, these studies used discrete methods in their investigation. In this study, coordination variability of the inter-segmental couplings was higher in specific parts of the task as compared to the healthy control group. This is in accordance with the results of previous investigations, which reported increased coordination variability in pathologic conditions [14,15,26].

The results of the present study demonstrated similar coordination patterns for the foot-shank coupling of the supporting leg between the two groups “Fig 3A”. This sounds reasonable because in our experiment right foot was bound flat on the step for a long duration after starting the task, so the foot might not have sufficient opportunity to show a variable behavior.

The results also demonstrated different coordination patterns of shank-thigh of the supporting leg in ACLD subjects compared to healthy control group “Fig 3B”. Compared to the control group, ACLD subjects had an in-phase relationship in roughly the first half of the task period, where the lowering of the body mass was being controlled by the supporting leg.

Since ACLD subjects demonstrated more in-phase relationship for both foot-shank and shank-thigh couplings in their supporting leg (i.e. foot, shank and thigh move in the same direction) “Fig 3A and 3B”, it seems that the body mass revolves around the distal component of the chain (i.e. the ankle joint) during forward continuance and controlled lowering phases of the task. This would transfer the control away from the unstable knee to the ankle, which may happen in ACLD subjects when they encounter challenging activities [30]. Coordination variability of the supporting foot-shank of the ACLD subjects was higher as compared to the healthy individuals especially around initial contact “Fig 3C”. By and large, coordination variability of the supporting foot-shank in both groups reached its highest value around initial contact, which may denote more challenging characteristics of this phase of the step-descent. This may be due to maximum divergence of the center of mass from center of pressure occurring at the end of the single-leg stance of the supporting leg [27]. For the shank-thigh coupling of the supporting leg, coordination variability showed a difference, which is more variable in the early part of the task for the patient group “Fig 3D”. This period is in accordance with the controlled lowering phase, which is challenging for ACLD subjects. The graph also showed more variability in ACLD subjects at the initial contact of the step-descent. Initial contact is a critical point in step-descent, which entails more balance compensation [27], hence more variability.

For the stepping leg, coordination pattern of the foot-shank coupling indicated different patterns between the two groups “Fig 3E”. These differences were most pronounced in the first 60 percent of the task period, where the ACLD subjects showed a more in-phase relationship before initial contact of the stepping leg. This is confirmed by prior research, which revealed altered forefoot strategy of the sound side during step descent task [16,31].

Regarding the stepping shank-thigh coordination pattern, the results demonstrated similar pattern between the two groups “Fig 3F”. This was inferred based on the coefficients of nonlinear regression analysis. The mentioned similarities may be due to compensation at the distal link, i.e. the foot-shank coupling which copes with the disabling effects of the injured leg.

Coordination variability of the foot-shank coupling of the stepping leg demonstrated differences between the two groups “Fig 3G”. This difference was more distinguished around initial contact, where higher variability for ACLD subjects was evident. The stepping shank-thigh coupling coordination variability showed difference so that the patient group demonstrated greater variability prior to the contact of the stepping leg “Fig 3H”. Increased variability may be a compensatory mechanism adopted by the central nervous system to control body descending in such a demanding task [14]. This increased coordination variability may also be due to the unstable nature of the knee and lack of afferent information of ACLD subjects [32]. Whether this increased variability is attributable to the unstable nature of ACLD subject (bad variability) or to the flexibility-stability (good variability) of the neuromusculoskeletal system is yet to be determined. Since such a different coordination pattern and variability persists even after reconstruction surgery [2,10], neuromuscular training is suggested to be included during rehabilitation program.

It this study, altered coordination pattern and coordination variability was evident for healthy left leg. These alterations may be attributed to the crossover caused by central nervous system which may affect bilateral balance [33] and quadriceps activation [34] in unilateral ACL rupture. This result confirms the results of the previous investigations which stated that sound side of the ACLD subjects is not a reliable standard for comparison to the injured side [4]. Therefore, it is suggested to use the term ‘less-affected’ side instead of ‘non-affected’ or intact side to denote non-injured side in ACLD subjects.

In the present study, kinematics of the hip, knee and ankle of both supporting and stepping legs did not show any significant difference between the healthy and the ACLD subjects. This is in accordance with previous studies which denoted spared kinematics of the lower limb in these patients during step descent task. This may be due to more knee flexion angle of the supporting leg during step descent activities as compared to other functional activities such as level walking [5]. In this regard, it could be suggested that during step activity, kinematic verifications would not be helpful in differentiating ACLD from healthy subjects. Rather, a more sophisticated dynamic approach such as CRP would uncover such discrepancies. In this study, kinematic data were examined at the early and at initial contact of the task period. We considered these instances because of challenging characteristics of the step task at these two points.

As stated earlier, the reversals in coordination pattern demonstrate changes in coordination dynamics. Such reversals represent a critical point in the task cycle [24]. In this study, coordination patterns of the supporting foot-shank and stepping shank-tight showed a reversal around 60 percent of the task. Likewise, coordination patterns of the supporting shank-thigh and stepping foot-shank showed a reversal around 70 percent of the task. These points correspond to the initial contact of the step-descent task, where body weight transfers from the supporting leg to the stepping leg and is considered a critical point “Dotted lines in Fig 3A–3F and 3B–3E”, respectively.

Preferred speed was not significantly different between the two groups, so the disparity in coordination pattern and coordination variability would not be attributable to the difference in stepping velocity of the task (Table 1).

Previous studies indicated that the time elapsed since injury may affect compensatory adaptations of the ACLD subject [7,35]. In this study, the duration of injury was in a narrow range, 8.02 ±2.28 months, resulting in more homogenous ACLD subjects.

ACLD subjects demonstrated different strategies in both lower extremities during a single-step descent. They also showed increased coordination variability, especially before the stepping leg contacts the floor. However, kinematic data did not show any difference between the two groups. Therefore, our results suggest that examining the coordination patterns of the limbs might better explain the pathobiomechanics of the ACLD subjects during step descent task.

## Supporting Information

S1 Data Mean ensemble coordination pattern and coordination variability. (XLSX)

S2 Data Ankle, knee and hip joint angles. (XLSX)

S3 Data Foot, shank and thigh segment angles. (XLSX)

S1 Fig Laboratory set-up, marker setting and initial position. (TIF)

## References

[1] Ferber R, Osternig LR, Woollacott MH, Wasielewski NJ, Lee J-H. Gait mechanics in chronic ACL deficiency and subsequent repair. Clinical biomechanics. 2002;17(4):274–85. 12034120 10.1016/s0268-0033(02)00016-5

[2] Kiefer AW, Ford KR, Paterno MV, Schmitt LC, Myer GD, Riley MA, et al Inter-segmental postural coordination measures differentiate athletes with ACL reconstruction from uninjured athletes. Gait & Posture. 2013;37(2):149–53. 23219784 10.1016/j.gaitpost.2012.05.005 PMC3556179

[3] Ferber R, Osternig LR, Woollacott MH, Wasielewski NJ, Lee J-H. Bilateral accommodations to anterior cruciate ligament deficiency and surgery. Clinical biomechanics. 2004;19(2):136–44. 14967576 10.1016/j.clinbiomech.2003.10.008

[4] Ingersoll C, Grindstaff T, Pietrosimone B, Hart J. Neuromuscular Consequences of Anterior Cruciate Ligament Injury. Clinics in sports medicine. 2008;27(3):383–404. 10.1016/j.csm.2008.03.004 18503874

[5] Berchuck M, Andriacchi TP, Bach BR, Reider B. Gait adaptations by patients who have a deficient anterior cruciate ligament. The Journal of Bone and Joint Surgery. 1990;72(6):871–7. 2365720

[6] Devita P, Hortobagyi T, Barrier J, Torry M, Glover KL, Speroni DL, et al Gait adaptations before and after anterior cruciate ligament reconstruction surgery. Medicine and science in sports and exercise. 1997;29(7):853–9. 9243483 10.1097/00005768-199707000-00003

[7] Wexler G, Hurwitz DE, Bush-Joseph CA, Andriacchi TP, Bach BR Jr. Functional gait adaptations in patients with anterior cruciate ligament deficiency over time. Clinical orthopaedics and related research. 1998;348:166–75. 9553549

[8] Barela JA, Whitall J, Black P, Clark JE. An examination of constraints affecting the intralimb coordination of hemiparetic gait. Human Movement Science. 2000;19(2):251–73.

[9] Seay JF, Van Emmerik RE, Hamill J. Low back pain status affects pelvis-trunk coordination and variability during walking and running. Clinical biomechanics. 2011;26(6):572–8. 10.1016/j.clinbiomech.2010.11.012 21536356

[10] Kurz MJ, Stergiou N, Buzzi UH, Georgoulis AD. The effect of anterior cruciate ligament recontruction on lower extremity relative phase dynamics during walking and running. Knee Surgery, Sports Traumatology, Arthroscopy. 2005;13(2):107–15. 15756615 10.1007/s00167-004-0554-0

[11] Hamill J, van Emmerik RE, Heiderscheit BC, Li L. A dynamical systems approach to lower extremity running injuries. Clinical biomechanics. 1999;14(5):297–308. 10521606 10.1016/s0268-0033(98)90092-4

[12] Hart JM, Ko J-WK, Konold T, Pietrosimione B. Sagittal plane knee joint moments following anterior cruciate ligament injury and reconstruction: A systematic review. Clinical biomechanics. 2010;25(4):277–83. 10.1016/j.clinbiomech.2009.12.004 20097459

[13] Button K, Vandeursen R, Price P. Recovery in functional non-copers following anterior cruciate ligament rupture as detected by gait kinematics. Physical Therapy in Sport. 2008;9(2):97–104. 10.1016/j.ptsp.2008.03.001 19083709

[14] Crowther RG, Spinks WL, Leicht AS, Quigley F, Golledge J. Intralimb coordination variability in peripheral arterial disease. Clinical biomechanics. 2008;23(3):357–64. 18061322 10.1016/j.clinbiomech.2007.10.009

[15] Van Uden C, Bloo J, Kooloos J, Van Kampen A, De Witte J, Wagenaar R. Coordination and stability of one-legged hopping patterns in patients with anterior cruciate ligament reconstruction: preliminary results. Clinical biomechanics. 2003;18(1):84–7. 12527252 10.1016/s0268-0033(02)00170-5

[16] Rudolph KS, Snyder-Mackler L. Effect of dynamic stability on a step task in ACL deficient individuals. Journal of Electromyography and Kinesiology. 2004;14(5):565–75. 15301775 10.1016/j.jelekin.2004.03.002

[17] Briggs KK, Kocher MS, Rodkey WG, Steadman JR. Reliability, validity, and responsiveness of the Lysholm knee score and Tegner activity scale for patients with meniscal injury of the knee. The Journal of Bone & Joint Surgery. 2006;88(4):698–705. 16595458 10.2106/JBJS.E.00339

[18] Richards J. Biomechanics in clinic and research: an interactive teaching and learning course Philadelphia, PA: Churchill Livingstone/Elsevier; 2008 p. 181.

[19] Hsu WL, Scholz JP. Motor abundance supports multitasking while standing. Human Movement Science. 2011;31(4):844–62. 10.1016/j.humov.2011.07.017 22094118 PMC3288691

[20] Lark SD, Buckley JG, Jones DA, Sargeant AJ. Knee and ankle range of motion during stepping down in elderly compared to young men. European journal of applied physiology. 2004;91(2):287–95. 14586586 10.1007/s00421-003-0981-5

[21] Andriacchi T, Andersson G, Fermier R, Stern D, Galante J. A study of lower-limb mechanics during stair-climbing. The Journal of Bone and Joint Surgery. 1980;62(5):749 7391098

[22] Hamill J, Heiderscheit BC, Pollard CD. Gender differences in lower extremity coupling variability during an unanticipated cutting maneuver. Journal of Applied Biomechanics. 2005;21(2):143–52. 16082015 10.1123/jab.21.2.143

[23] Heiderscheit BC, Hamill J, van Emmerik RE. Variability of stride characteristics and joint coordination among individuals with unilateral patellofemoral pain. Journal of Applied Biomechanics. 2002;18(2):110–21.

[24] Hamill J, McDermott WJ, Haddad JM. Issues in quantifying variability from a dynamical systems perspective. Journal of Applied Biomechanics. 2000;16(4):407–18.

[25] Wilson C, Simpson S, Van Emmerik R, Hamill J. Coordination variability and skill development in expert triple jumpers. Sports Biomechanics. 2008;7(1):2–9. 10.1080/14763140701682983 18341132

[26] Chiu S-L, Lu T-W, Chou L-S. Altered inter-joint coordination during walking in patients with total hip arthroplasty. Gait & Posture. 2010;32(4):656–60. 20947354 10.1016/j.gaitpost.2010.09.015

[27] Zachazewski JE, Riley PO, Krebs DE. Biomechanical analysis of body mass transfer during stair ascent and descent of healthy subjects. Journal of rehabilitation research and development. 1993;30:412–22. 8158557

[28] Knoll Z, Kocsis Ls, Kiss RM. Gait patterns before and after anterior cruciate ligament reconstruction. Knee Surgery, Sports Traumatology, Arthroscopy. 2004;12(1):7–14. 14586491 10.1007/s00167-003-0440-1

[29] Gao B, Cordova ML, Zheng N. Three-dimensional joint kinematics of ACL-deficient and ACL-reconstructed knees during stair ascent and descent. Human Movement Science. 2011;32(1):222–35. 10.1016/j.humov.2011.04.009 21798608

[30] Hurd WJ, Snyder Mackler L. Knee instability after acute ACL rupture affects movement patterns during the mid stance phase of gait. Journal of Orthopaedic Research. 2007;25(10):1369–77. 17557321 10.1002/jor.20440 PMC2859715

[31] Rudolph K, Eastlack M, Axe M, Snyder-Mackler L. Basmajian Student Award Paper: Movement patterns after anterior cruciate ligament injury: a comparison of patients who compensate well for the injury and those who require operative stabilization. Journal of Electromyography and Kinesiology 1998;8(6):349–62. 9840891 10.1016/s1050-6411(97)00042-4

[32] Stergiou N, Harbourne RT, Cavanaugh JT. Optimal movement variability: a new theoretical perspective for neurologic physical therapy. Journal of Neurologic Physical Therapy. 2006;30(3):120–9. 17029655 10.1097/01.npt.0000281949.48193.d9

[33] Lysholm M, Ledin T, Ödkvist L, Good L. Postural control—a comparison between patients with chronic anterior cruciate ligament insufficiency and healthy individuals. Scandinavian Journal of Medicine & Science in Sports. 1998;8(6):432–8. 9863982 10.1111/j.1600-0838.1998.tb00464.x

[34] Chmielewski TL, Stackhouse S, Axe MJ, Snyder‐Mackler L. A prospective analysis of incidence and severity of quadriceps inhibition in a consecutive sample of 100 patients with complete acute anterior cruciate ligament rupture. Journal of Orthopaedic Research. 2004;22(5):925–30. 15304261 10.1016/j.orthres.2004.01.007

[35] Kuster M, Sakurai S, Wood G. The anterior cruciate ligament-deficient knee: compensatory mechanisms during downhill walking. The Knee. 1995;2(2):105–11.

