# Supplementary figures and images for: Inter-Segmental Coordination Pattern in Patients with Anterior Cruciate Ligament Deficiency during a Single-Step Descent
Source: PLoS One. 2016 Feb 22;11(2):e0149837. doi: 10.1371/journal.pone.0149837 (PMC4762765; doi:10.1371/journal.pone.0149837)

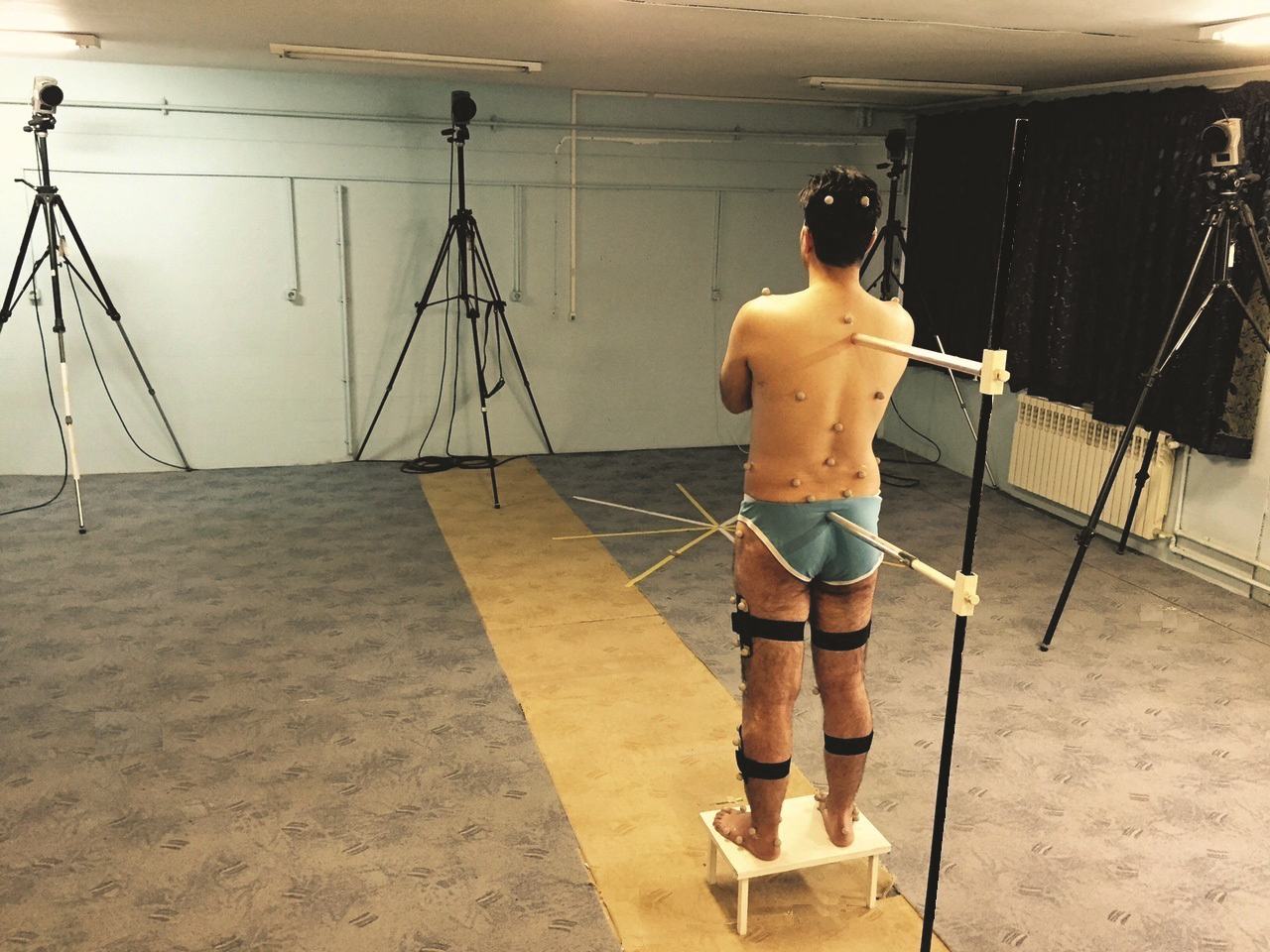

Supplement: S1 Fig — (TIF) [file pone.0149837.s004.TIF]
